# Development and validation of a reading comprehension and fluency screening assessment tool for children aged 7–10: implications for audiological rehabilitation

**DOI:** 10.3389/fpsyg.2026.1759333

**Published:** 2026-02-04

**Authors:** Bahtiyar Çelikgün, Ferda Akdaş, Büşra Nur Eser, Recep Minga, Gül Ölçek, Cansu Gücüyener, Sude Edman

**Affiliations:** 1 School of Health Sciences, Department of Audiology, Istanbul Medipol University, Istanbul, Türkiye; 2 Audiology Clinic, Istanbul Academic Hospital, Istanbul, Türkiye; 3 Graduate School of Social Sciences, Management and Strategy, Istanbul Medipol University, Istanbul, Türkiye

**Keywords:** psychometrics, reading, reading comprehension, reading fluency, writing

## Abstract

This study presents a valid and reliable Turkish Reading Comprehension and Fluency Screening Assessment Tool (RCF-SAT) for children aged 7 to 10, which was developed and standardized. Considering the absence of practical, audiologist-friendly instruments in existing literature, this study introduces an original screening assessment tool that evaluates reading comprehension, reading speed, and writing fluency. Consisting of a short narrative passage and eight open-ended questions, RCF-SAT was administered to a diverse sample of 457 primary school students. Statistical analyses, including explanatory and confirmatory factor analyses, validated the RCF-SAT’s structure. Reliability coefficients (Cronbach’s *α* = 0.861; McDonald’s *ω* = 0.859) confirmed their internal consistency. The results showed consistent improvements in reading and writing performance with age. This tool provides a quick, accessible, and statistically robust method for evaluating literacy development in Turkish-speaking children.

## Introduction

Reading and writing skills, which are fundamental components of communication skills, play a critical role in the cognitive, academic, and social life of the individual. The acquisition of phonetic, syntactic, and semantic structures of the language, particularly in early childhood, is of paramount importance for the development of reading comprehension skills (Hjetland et al., 2019). Moreover, the development of reading comprehension skills is not confined to the process of decoding written text. According to the Simple View of Reading (SVR) model, reading comprehension (RC) skill is the product of word-decoding (D) and linguistic comprehension (LC) components. When any of these two components is low, total reading success is also limited to the same extent (formalized as RC = D × LC) (Hoover and Gough, 1990). Beyond the two-component framework proposed by the Simple View of Reading, successful comprehension also requires metacognitive processes such as meaning construction, inference generation, and the integration of information across sentences. Accordingly, reading comprehension should be conceptualized as a multidimensional skill that emerges from the dynamic interaction of cognitive and linguistic processes.

The Turkish orthographic system is transparent and phonologically consistent, which enables early readers to attain high decoding accuracy by the end of Grade 1. However, reading fluency at the level of connected text continues to develop throughout the upper elementary grades, suggesting that decoding skills plateau early while linguistic comprehension becomes the primary constraint on reading proficiency (Guzel Ozmen, 2011).

This developmental shift places children with hearing loss at a particular disadvantage, as limited access to auditory input may undermine the oral language foundations required for higher-level text comprehension. Oral and written communication processes exhibit a high degree of interdependence. Children with hearing loss frequently exhibit an underdeveloped phonological representation, a deficiency that arises from diminished early auditory stimulation. This impairment impedes both the accuracy of decoding and the comprehension of higher-order linguistic structures (McGuckian and Henry, 2007; Pittman et al., 2005). Consequently, these children encounter persistent disadvantages in acquiring critical linguistic structures, such as syntax, function words, and morphological cues (McGuckian and Henry, 2007; Nittrouer and Burton, 2005). Such fundamental deficiencies frequently result in diminished reading comprehension and fluency in comparison to their hearing peers (Camarata et al., 2018; Park et al., 2013).

When oral and written communication skills are considered as a whole, it is imperative for audiologists to monitor the development of children with hearing loss in both communication styles. Conversely, measurement tools such as the Woodcock Reading Mastery Test (WRMT) and the Woodcock-Johnson Test of Achievement (WJ V), which are widely employed internationally, are constrained in terms of practical application due to their predominantly complex subtests and extended application time (Bradley-Johnson et al., 2004; De Rose, 1999). The utilization of multiple texts in fluency assessments, such as the Gray Oral Reading Test, has been shown to increase the time and resource demands on practitioners.

In the context of Türkiye, a notable proportion of reading comprehension assessments are meticulously crafted by educators (Kaya et al., 2018; Kökçü and Demirel, 2020; Çetinkaya and Akyol, 2019; Ülper et al., 2017). These assessments are predominantly designed for specific age groups, particularly those in the fourth grade and above, and frequently encompass multiple-choice inquiries. It is evident that the existing assessments do not possess a structural design that encompasses the evaluation of reading and writing fluency, in addition to reading comprehension skills, in a holistic manner. Furthermore, these assessments lack the provision of norm data that is specific to each age level, thereby hindering their applicability in clinical settings.

Consequently, there is a clear need to develop a standardized evaluation instrument that can comprehensively assess reading comprehension and reading fluency in children aged 7 to 10 years while providing age-specific normative data. Such an instrument should also be sensitive to auditory performance and suitable for clinical use by audiologists working with children with hearing loss. Early identification of reading comprehension difficulties is particularly important to prevent long-term academic underachievement, particularly in children who demonstrate adequate decoding skills but subtle comprehension weaknesses. In many educational systems, including that of Türkiye, the limited availability of time-efficient assessment tools restricts early detection. A practical and standardized screening assessment tool may therefore support both clinicians and educators in identifying children at risk for delayed literacy development or learning difficulties.

Despite this critical need, there is currently no standardized assessment tool in Turkish that simultaneously evaluates reading comprehension, reading fluency, and writing performance in children, regardless of hearing status. Existing tools are either limited to specific age groups, focus solely on comprehension accuracy, or lack normative data suitable for clinical use Therefore, the primary aim of this study was to develop and standardize a Turkish RCF-SAT for children aged 7–10 years. In addition to establishing normative data, this study aimed to examine the psychometric properties of the test and to highlight its potential utility as a practical screening instrument in audiological and educational settings.

## Materials and methods

### Development of materials

RCF-SAT was designed to be both pragmatic and time-efficient while capturing key components of the comprehension process. Given the age range of the target population, tasks such as summarizing the text, identifying the main idea, or generating titles were considered developmentally demanding and were therefore excluded. Instead, the reading passage and accompanying questions were constructed to assess comprehension through concrete and clearly identifiable textual information. Therefore, the reading text entitled “My Grandfather,” which was utilized as the “reading passage” in the developed tool, was meticulously designed to incorporate the semantic, syntactic, and related characteristic structures of the language. Furthermore, the content of the text was prepared in a balanced way, considering individual differences and cognitive capacity between the ages of 7 and 10.

The passage under consideration consists of a total of 127 words and 17 meaningful sentences. The paragraph, which conveys the perspective of a grandchild on her grandfather, was crafted to be as straightforward and accessible as possible. Participants were instructed to first read the paragraph and subsequently read the 8 questions prepared according to the paragraph and answer them in writing. The participants’ reading and writing times were documented.

During the preparation of the questions, adjectives describing the physical characteristics of the grandfather (e.g., blue eyes, gray hair), trees in the grandfather’s garden (e.g., oranges, lemons, and bananas), or foods that the grandfather purchased for his grandchild (e.g., cotton candy and bagels) were requested. The objective was to formulate responses to these inquiries that were both concise and unambiguous. A subsequent analysis of the eight responses revealed that five of them consisted of a single item. For instance, one inquiry pertained to the color of the grandfather’s eyes, with potential responses encompassing “blue.” The correct responses to two questions comprised three items, such as the identification of other fruit trees present in the garden, besides pears, oranges, lemons, and bananas. The response to the question was comprised of two items: the specific foods that his grandfather purchased for her in the park, identified as cotton candy and bagels. The participants were instructed to respond to the questions by articulating the expressions they desired.

While the present study was chiefly concerned with the validation of RCF-SAT, the administration procedure was conceived with the objective of ensuring its feasibility for large groups in school settings, thereby paving the way for future applications in educational screening and early intervention contexts.

The researchers also documented the writing process using a stopwatch. Consequently, the duration required for each participant to complete the reading passage and respond to the inquiries was meticulously documented.

### Pilot study

Prior to the implementation of the RCF-SAT on a large population, five experts provided their insights into the paragraphs and questions. These insights were subsequently integrated into the pilot study, wherein the paragraphs and questions underwent revisions. A total of 16 children with normal hearing, with four children from each class ranging in age from seven to 10 years old, participated in the pilot study. In the pilot study, participants were assigned to read the reading passage entitled “My Grandfather” and answer five questions prepared about the passage. The duration of each reading and the time required to respond to each question were measured and recorded with a stopwatch. Subsequent to the pilot study, the reading passage and the questions asked underwent revisions, and the “RCF-SAT” was finalized. The reading passage employed in the pilot study consisted of 20 sentences and 121 words. The text resulting from the revision process contained 127 words and 17 sentences. The initial iteration of the questionnaire comprised five questions; however, this number was augmented to eight in the final version.

### Ethics statement

The study protocol was reviewed and approved by the İstanbul Medipol University Non-Interventional Clinical Research Ethics Committee (Approval No: E-10840098-772.02-5577, Date: 04/09/2023). Written parental consent and child assent were obtained in accordance with the Declaration of Helsinki.

### Participants

The study was conducted with a total of 457 students from the first, second, third, and fourth grades of primary school. The distribution of gender, grade, and socioeconomic level were identified as key factors. The socioeconomic level of the participants was determined according to the average housing rent values of the districts in Istanbul and the private/public school category. Accordingly, the study population was created using data collected from public schools in the two districts with the lowest housing rent averages, two public schools with average rent values, and a private school with a high rent value.

Socioeconomic status is widely recognized as a multidimensional construction that includes education, occupation, income, and access to resources. When individual socioeconomic data are not available, area-based indicators are commonly used as proxy measures, particularly in large urban studies. In metropolitan contexts such as Istanbul, district-level average housing rent values reflect both household economic capacity and neighborhood characteristics, including access to educational resources and social infrastructure (Mercan and Akyol, 2023). Accordingly, in the present study, average housing rent value was adopted as an indicator of socioeconomic status.

### Procedure

The study was conducted by two researchers who visited schools. Prior to conducting the study in the designated schools, permission was obtained from the Ministry of National Education and the institution administrators. Written parental consent and child assent were obtained prior to participation.

Students were randomly selected by the researchers and tested individually. Each participant was tested individually in a quiet room. Following standardized instructions, the child was asked to read the passage aloud and then answer the written questions. Reading time and written response time were measured using a stopwatch. Timing began at the researcher’s verbal start cue and ended when the participant indicated task completion.

In instances where the participants provided answers in the form of a single word or sentence, and if the aforementioned items corresponded to those listed in the answer key, the question was deemed to have been correctly answered. In multiple-item questions, each item was scored separately (e.g., orange, lemon, and banana were scored separately).

The data collection process was initiated in April 2024, contingent upon the adequate development of reading skills exhibited by the first graders. The collection process was concluded in June 2024.

A participant who provided all the anticipated responses received a perfect score of 13 on the RCF-SAT. In order to render the scoring system practical, the scoring was transitioned to a hundred-point system by a researcher who is an expert in the field of statistics. To facilitate the calculation of scores, each participant’s correctly answered questions were assigned a value of 7.7 points. Consequently, a participant who answered all 13 questions correctly would reach approximately 100 points. The normalization curves for each sub-test were converted into a tabular format. Consequently, the reading comprehension, reading, and writing fluency of each participant were converted into a practical normalization curve, which can be readily utilized by audiologists.

### Statistical analysis

The data obtained from 457 student participants in the study were analyzed using SPSS for Windows 25.00, Categorical Principal Component Analysis (CATPCA) 2.0, and Jamovi (2.3) programs. The “RCF-SAT,” which was developed for this study, underwent a discovery factor analysis to ascertain its dimensional structure. The reliability of the scale was ascertained through the implementation of two distinct statistical methodologies: Cronbach’s alpha and McDonald’s coefficient.

## Results

### Demographic characteristics of the participants

A total of 457 students enrolled in the first, second, third, and fourth grades of primary school participated in the study. The population of students was comprised of 219 (47.9%) female and 238 (52.1%) male students. The distribution of the students’ grades and socioeconomic levels is presented in Table 1.

**Table 1:** Demographic characteristics of the participants.

Variables		*N*	%
Gender	Female	219	47.9%
	Male	238	52.1%
Grade	1 (7 years old)	96	21.0%
	2 (8 years old)	109	23.9%
	3 (9 years old)	127	27.8%
	4 (10 years old)	125	27.4%
SEL*	Low	182	39.8%
	Average	163	35.7%
	High	112	24.5%

*SEL, Socioeconomic Level.

### Factor analysis of the scale

First, an explanatory factor analysis was performed on the “Turkish Reading Comprehension Scale” created for this study, and its dimensional structure was examined. The present study employed CATPCA (2.0), an add-on in IBM SPSS, in the execution of the explanatory (discovery) factor analysis. The rotation technique was implemented in conjunction with the Principal Normalization and Varimax method within the CATPCA 2.0 program. The factor loadings, explanatory ratio, and reliability value obtained in the single-factor structure of the scale are presented in Table 2.

**Table 2:** Summary of explanatory factor analysis.

Questions (Items)	Factor loadings	Explanatory (%)	Cronbach’s alpha	McDonald’s omega
Q1	0.524	%53.13	0.861	0.859
Q2	0.622			
Q3	0.609			
Q4	0.657			
Q5.1	0.562			
Q5.2	0.602			
Q5.3	0.722			
Q6.1	0.552			
Q6.2	0.652			
Q6.3	0.696			
Q7	0.716			
Q8.1	0.581			
Q8.2	0.569			

Variable principal normalization.

Prior to conducting the factor analysis, Bartlett’s sphericity test was conducted, yielding a *p*-value of less than 0.05. Additionally, the Kaiser–Meyer–Olkin (KMO) value was determined to be 0.882. According to these values, the data set is “perfectly” suitable for factor analysis. In the principal component analysis, which is identified as one dimension, it is evident that the factor loadings of the items range from 0.524 to 0.722. The explanatory power of the one-dimensional structure was found to be 53.13%. Given that the Cronbach’s alpha value was determined to be 0.861 in the CATPCA add-on —a measure of reliability and internal consistency of the scale— and the McDonald’s omega value was found to be 0.859 in the Jamovi program, it can be concluded that the scale is valid and reliable for the sample.

### Confirmatory factor analysis of reading comprehension score

In the confirmatory factor analysis, as the sample size increases -particularly in samples exceeding 200- the Chi-Square (*x^2^*) value increases and the statistical significance level of the Chi-Square (*x^2^*) test decreases. The evaluation of the scales utilized in the research study, as well as the general suitability of the models, was determined through a comprehensive examination of the *x^2^*distribution with degrees of freedom (*x^2^* value/df), additional goodness-of-fit indices, and the values in the standardized residual covariance matrix.

In the confirmatory factor analysis of the developed reading comprehension scale, the validity of the one-dimensional structure obtained from the explanatory factor analysis was tested. The results of the analysis indicated that the standard factor loadings ranged from 0.63 to 0.91, and the *p*-value was less than 0.05 for all items. The model parameters obtained from the confirmatory factor analysis are as follows: *x^2^* = 167.57 and *x^2^*/df = 2.578, *p* < 0.05. These findings suggest that the model is significant. Following a detailed examination of the model’s fit index values, it has been determined that the observed values (GFI = 0.947, CFI = 0.946, SRMR = 0.0442, RMSEA = 0.061) are within the established acceptable fit limits. This finding suggests that the model is valid (Table 3).

**Table 3:** Goodness-of-fit indexes and fit values used in confirmatory factor analysis.

Indexes	Goodness-of-fit	Acceptable compliance	Reading comprehension scale
*x*^2^/df	0 ≤ *x*^2^/df ≤ 2	2 < *x*^2^/df ≤ 3	2.578
GFI	≥ 0.90	0.85–0.89	0.947
CFI	≥ 0.97	≥ 0.95	0.946
SRMR	≤0.05	0.06 ≤ SRMR ≤ 0.08	0.0422
RMSEA	≤ 0.05	0.06 ≤ RMSEA ≤ 0.08	0.0611

### Normalization values of reading comprehension, reading, and writing speed

**Figure 1:**
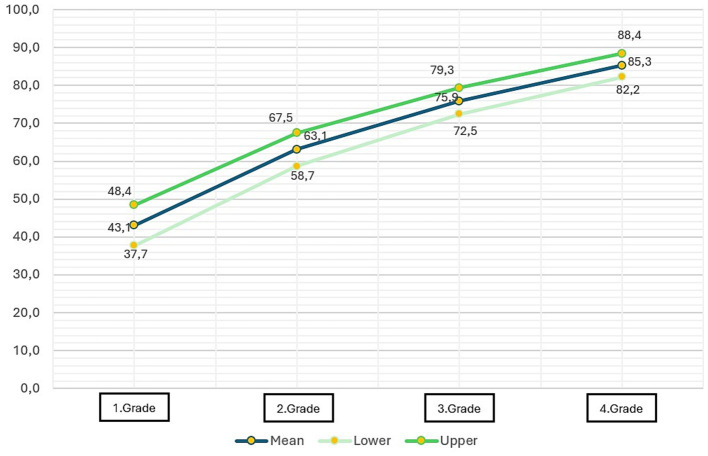
Mean, lower, and upper values of reading comprehension scores (out of 100 points).

An examination of the mean reading comprehension scores of the entire sample of participants reveals a consistent improvement in reading comprehension skills with both age and grade level. As demonstrated in Table 4 and Figure 1, the mean reading comprehension score in first grade is 43.07 out of 100. This value increases with age, reaching 63.07, 75.90, and 85.32, respectively.

**Table 4:** Mean, lower, and upper values of reading comprehension scores (out of 100 points).

Grade	Mean	SD	Lower	Upper
1	43.073	2.6966	37.719	48.426
2	63.073	2.2244	58.664	67.482
3	75.906	0.7350	72.472	79.339
4	85.320	1.5706	82.211	88.429

**Figure 2:**
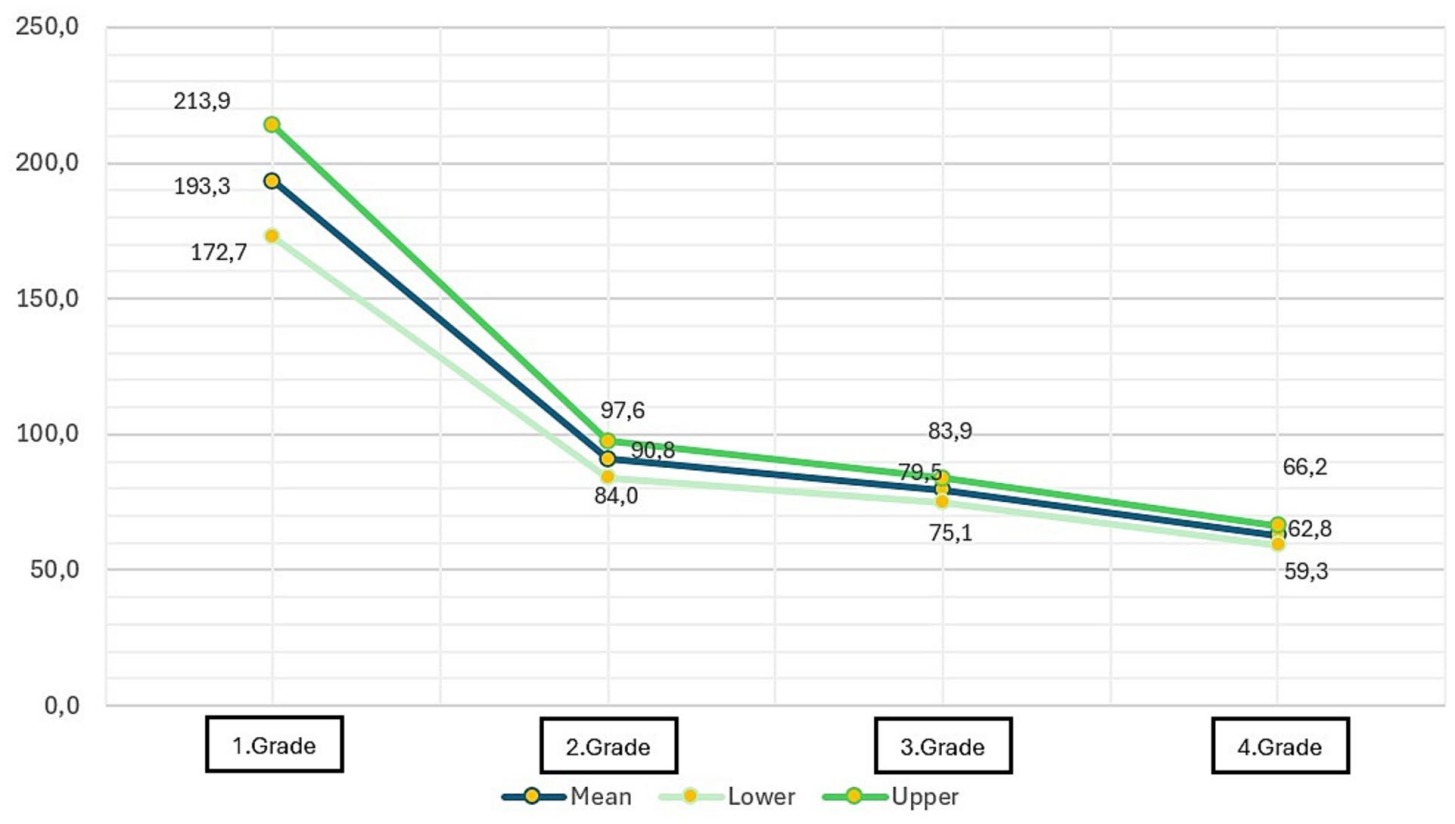
Mean, lower, and upper values of reading fluency scores (in terms of seconds).

The evaluation of reading fluency entailed the measurement of reading speed. As the reading fluency evaluation progressed, a consistent decrease in the time required to respond to questions was observed as the participants advanced in age. The salient point is that this development constitutes a substantial leap from first grade to second grade, while the increase in second, third, and fourth grades is not a dramatic increase. The mean reading speed of first grade students for the text “Dedem” is 193.30 s, whereas for second grade students, this value is 90.82. The values in third and fourth grades are 79.48 and 62.78, respectively (Table 5; Figure 2).

**Table 5:** Mean, lower, and upper values of reading fluency scores (in terms of seconds).

Grade	Mean	SD	Lower	Upper
1	193.3021	10.36986	172.7153	213.8889
2	90.8257	3.42697	84.0328	97.6185
3	79.4882	2.24218	75.0510	83.9254
4	62.7840	1.74794	59.3243	66.2437

**Figure 3:**
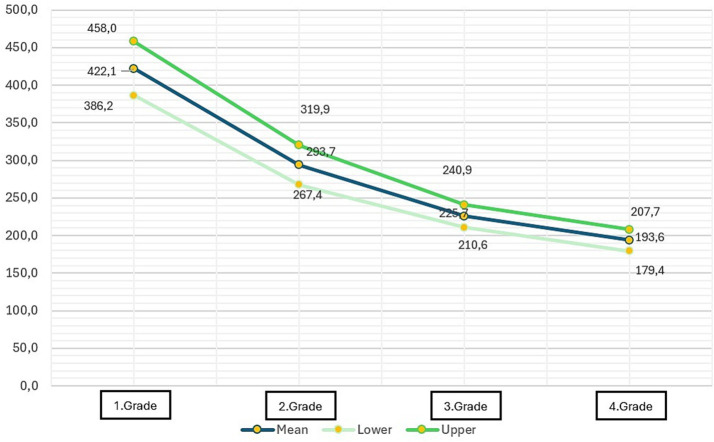
Mean, lower, and upper values of duration of writing answers to questions (in terms of seconds).

In the course of the evaluation of writing fluency, a consistent decrease in the answering times for questions was observed with increasing age. The mean response time, which was 422.10 s in the first grade, was calculated as 293.66, 225.72, and 193.56 s from the second to the fourth grades, respectively, as the grade increased (Table 6; Figure 3).

**Table 6:** Mean, lower, and upper values of duration of writing answers to questions (in terms of seconds).

Grade	Mean	SD	Lower	Upper
1	422.1042	18.08256	386.2057	458.0026
2	293.6697	13.23418	267.4373	319.9022
3	225.7244	7.65698	210.5715	240.8774
4	193.5680	7.14606	179.4239	207.7121

## Discussion

The interdependence between oral language skills and written literacy is well established; however, findings regarding reading outcomes in children with hearing loss remain inconsistent. While some studies report comparable reading comprehension skills between children with hearing loss and their normal-hearing peers, others document persistent difficulties in reading fluency and overall literacy performance (Camarata et al., 2018; Çelikgün and Akdaş, 2021; Park et al., 2013; Tomblin et al., 2020).

These discrepancies are likely attributable to differences in participant characteristics, including age at intervention, degree of hearing loss, effectiveness of auditory devices, family involvement, and variability in assessment methods across studies (Camarata et al., 2018; Park et al., 2013).

Within this context, the development of a new reading comprehension screening assessment tool represents an important contribution to the field. Existing reading assessments are often designed for broad educational purposes and may not adequately capture the specific literacy challenges experienced by children with hearing loss or those at risk for language-based difficulties. Moreover, many widely used instruments are time-consuming, rely heavily on multiple-choice formats, or lack sensitivity to subtle comprehension weaknesses, particularly in children who demonstrate adequate decoding skills. The present RCF-SAT was developed to address these limitations by integrating reading comprehension, fluency, and writing performance within a single, time-efficient framework, thereby offering a clinically meaningful alternative for use in audiological and interdisciplinary settings.

Beyond participant-related audiological factors, the choice of assessment instruments also influences reported reading outcomes. Many widely used reading tests were developed primarily for educational or speech-language contexts and reflect profession-specific priorities. For example, the Woodcock Reading Mastery Test-III (Woodcock, 1973) covers a broad age range but requires 15–45 min and includes complex subtests, limiting its practicality for audiological screening. Concerns regarding its reliability have also been reported (De Rose, 1999). Similarly, the Woodcock-Johnson Tests of Achievement (Woodcock et al., 2001), although broadly applicable, are often criticized for their length and technical variability. The Gray Oral Reading Test, currently in its fifth edition (Wiederholt and Bryant, 2012), requires multiple booklets and passages and may be resource-intensive in routine clinical settings.

Nationally, Turkish reading comprehension assessments are typically developed for educational contexts. For example, Ülper et al. (2017) administered a 28-item mixed-format test to 636 students across six regions. Kaya et al. (2018) designed a 32-item multiple-choice test for fourth graders. Çetinkaya and Akyol (2019) developed a 36-item multiple-choice test for fourth-grade students from different socioeconomic backgrounds. Kökçü and Demirel (2020) created narrative and informative comprehension tests for secondary school students. While valuable, these tools differ substantially from the present study in format, grade level, and inclusion of fluency measures.

The RCF-SAT demonstrates distinct advantages: (1) a heterogeneous sample representing multiple socioeconomic levels, (2) normalization for each primary grade, (3) integration of reading comprehension, fluency, and writing within eight open-ended items, and (4) a completion time of under 10 min. Such features enhance its clinical usability, particularly for audiologists.

Beyond its immediate clinical applications, the RCF-SAT may also function as a screening instrument for the early identification of children at risk for reading or learning difficulties. In collaboration with educational professionals, the test may support timely referral and intervention decisions by providing a rapid and objective measure of reading comprehension. Although the tool was developed in Turkish, its structured format and scoring approach may allow for adaptation to other languages following appropriate linguistic and cultural validation.

Despite its potential clinical utility, the interpretation of the present findings requires consideration of certain methodological limitations. First, the RCF-SAT is based on a single reading passage and open-ended questions; although alternative formats may offer broader assessment coverage, the scope of the tool was intentionally restricted to ensure practicality. Second, the sample was drawn from a single metropolitan area. While Istanbul’s demographic diversity partly mitigates this limitation, future studies should include broader national samples. Finally, because the RCF-SAT was developed in Turkish, its direct application in other languages is limited and would require language-specific adaptation. Given that English-speaking countries themselves vary widely in their educational systems, any cross-cultural adaptation will require revisions tailored to each national context rather than a one-size-fits-all model.

From a clinical perspective, it is important to note that the present study included only children with normal hearing. Future validation studies should incorporate children with varying degrees of hearing loss to establish hearing-specific norms. In addition, the writing fluency measure may partly reflect fine-motor skills rather than purely linguistic ability, which should be considered in interpretation. Practical challenges in audiological rehabilitation—such as ensuring quiet testing environments, managing amplification devices, and accounting for auditory fatigue—must also be examined in subsequent applications.

For future international applications, a clear methodological framework will be necessary. Adaptation should involve rigorous translation and back-translation to preserve meaning, cultural modification of story elements to ensure familiarity for children, and adjustment of text length and difficulty according to the developmental standards of each educational system. Psychometric validation, including reliability, factor analysis, and normative data, must be repeated in the target population. Finally, pilot testing in small samples followed by large-scale norming will be essential to establish the test’s validity and usability across languages and cultural contexts.

### Clinical implications

The RCF-SAT developed in the present study offers several practical advantages for clinical practice. It is based on a heterogeneous sample representing different socioeconomic levels and provides grade-specific normative data for children between 7 and 10 years of age. The RCF-SAT integrates reading comprehension, reading fluency, and writing performance within a single, brief assessment that can be completed in under 10 mins, enhancing its feasibility for routine clinical use by audiologists.

By comparing an individual child’s scores with the normative data established in this study, clinicians can identify early signs of literacy difficulties and plan targeted interventions, such as individualized auditory training, speech-language therapy, or referral to educational specialists. The open-ended question format not only quantifies comprehension and fluency but also provides qualitative insights into expressive language abilities, which are often missed in multiple-choice assessments. In this respect, the findings indicate that the RCF-SAT is capable of reliably distinguishing between different levels of reading comprehension ability. This is a prerequisite for its potential use in early identification contexts.

Beyond audiological practice, the RCF-SAT may also be informative for speech-language pathologists and auditory verbal therapists who work with children with hearing loss. For speech-language pathologists, the open-ended response format provides valuable insight into expressive language skills, syntactic organization, and narrative coherence, which are critical targets in language intervention. For auditory verbal therapists, repeated administration of the tool may support the monitoring of auditory-based language development and literacy-related outcomes over time. The brief administration time and integrated assessment of comprehension and fluency enhance the feasibility of using the tool across different clinical and educational settings.

While the present study was conducted with normal hearing children, the RCF-SAT was designed to be sensitive to auditory performance. Subsequent studies should validate its use in children with different degrees and types of hearing loss to establish clinical norms. This will enable the test to become a valuable addition to comprehensive rehabilitation programs, supporting audiologists in monitoring both auditory and literacy outcomes in children.

## Conclusion

A comprehensive study was conducted to assess the reading comprehension, reading, and writing speeds of students in grades one, two, three, and four of primary school (ages seven, eight, nine, and ten, respectively). The study employed a heterogeneous sample of participants, ensuring uniformity in age, gender, and socioeconomic status. This practical screening assessment tool, which consists of a short reading passage and eight questions about the passage, has high statistical validity and reliability. The evaluation of the developed RCF-SAT was facilitated with a 100-point score system, and the evaluation of the results was made quite practical with the “normalization graphics” shown in the study. The RCF-SAT which was developed as a result of the study, has been shown to be a valid and reliable assessment instrument. This evaluation method is particularly well-suited for implementation by audiologists. In clinical practice, this test may serve as a fast-screening assessment tool for audiologists to track literacy outcomes in children with hearing loss.

In conclusion, the RCF-SAT’s psychometric robustness is noteworthy, and its practical application in early educational screening within the Turkish context is significant. Subsequent studies ought to assess the sensitivity and specificity of the method in identifying students at risk for developmental or learning difficulties, with a focus on both children with normal hearing and those with hearing loss.

## Data Availability

The raw data supporting the conclusions of this article will be made available by the authors, without undue reservation.
